# *Cellulosimicrobium cellulans* aortic prosthetic valve endocarditis

**DOI:** 10.1099/acmi.0.000068

**Published:** 2019-10-21

**Authors:** Jacopo Monticelli, Riccardo Gerloni, Claudio Farina, Anna Knezevich, Franca Dore, Roberto Luzzati

**Affiliations:** ^1^​ SC Malattie Infettive, Azienda Sanitaria Universitaria Integrata di Trieste, Trieste, Italy; ^2^​ SC Medicina d’Urgenza, Azienda Sanitaria Universitaria Integrata di Trieste, Trieste, Italy; ^3^​ UOC Microbiologia e Virologia, ASST ‘Papa Giovanni XXIII’, Bergamo, Italy; ^4^​ SC Laboratorio Analisi, Azienda Sanitaria Universitaria Integrata di Trieste, Trieste, Italy; ^5^​ SC Medicina Nucleare, Azienda Sanitaria Universitaria Integrata di Trieste, Trieste, Italy

**Keywords:** *Cellulosimicrobium*, endocarditis, single photon emission computed tomography

## Abstract

**Introduction.:**

Invasive infections due to *Cellulosimicrobium* spp. (a Gram-positive coryneform) are extremely rare. Only a few cases of bloodstream infections and endocarditis have been described, as bacteraemia due to coryneforms is usually discarded as blood culture contamination.

**Case presentation.:**

A 66-year-old female, with a history of aortic valve replacement, presented with fever, left leg purpura and acute kidney injury. Multiple repeated blood cultures were positive for *Cellulosimicrobium cellulans *, and targeted therapy was started. At first, endocarditis was excluded by echocardiograms, and the acute nephritis was interpreted as an atypical presentation of Henoch–Shönlein purpura. High-dose prednisone was started, and after 10 weeks the patient presented again with fever, mental confusion and acute left arm ischaemia. A subsequent echocardiogram and radiolabelled leukocyte scintigraphic evaluation revealed aortic prosthetic valve endocarditis with periprosthetic abscess and arterial brachial thrombosis. The patient deceased, and the autoptic examination confirmed an aortic valve periprosthetic abscess and revealed multiple arterial thromboses and septic embolisms in the kidneys, brain, spleen and myocardium.

**Conclusion.:**

Isolation of coryneform bacteria on blood culture should not always be discarded as blood culture contamination. In the case of endocarditis due to *Cellulosimicrobium* spp., the removal of any prosthetic material, along with prolonged *in vitro* active antimicrobial therapy, should be pursued in order to reduce persistence or relapses of infection.

## Case report

A 66-year-old female presented to the emergency room of our hospital for suspected left leg deep vein thrombosis (day 1). Her past medical history included aortic valve replacement with a sutureless bioprosthesis for severe valve stenosis 2 years before, and chronic gastritis. The patient complained of fever and pain in the left ankle 5 days before the present hospital admission. Her left leg was purpuric and painful, and the patient was febrile (37.5 °C). However, the venous Doppler scan was negative. Following admission to an internal medicine ward, routine laboratory analyses were normal with the exception of microhaematuria (26 erythrocytes μl^−1^ – normal values <10), intense haemoglobinuria and mildly elevated serum creatinine (0.11 mmol l^−1^ – normal values 0.03–0.09). At day 2, blood cultures were drawn from peripheral veins, and these were negative. Fever and left leg purpura endured and, on day 7, empirical antibiotic therapy with vancomycin and meropenem was initiated. On day 12 two blood cultures were repeated from peripheral veins and, after 48 h of incubation, both specimens were positive for pleomorphic, filamentous and branching Gram-positive rods. Both transthoracic (TTE) and transesophageal (TEE) echocardiograms were negative for echographic signs of endocarditis (day 17). The patient remained febrile and on days 19 and 28 blood cultures from peripheral veins were repeated. On a single blood culture set (anaerobes and aerobes) performed on day 28, the same Gram-positive rods grew again. Even if both samples from day 19 were negative, the day 28 blood cultures were again positive for Gram-positive rods. Subcultures were positive on 5 % sheep blood agar incubated aerobically and on chocolate plates incubated in 5 % CO_2_ atmosphere, where 2 mm shiny yellow-pigmented colonies were evident after 18 h of incubation. However, they were unidentified by Vitek2 (bioMérieux, France). Further, matrix-assisted laser desorption ionization/time-of-flight (MALDI-TOF) analysis (Vitek MS, bioMérieux, France) was unable to identify the organism. Since none of the phenotypical tests were able to provide correct identification of the strain, genotypical identification was performed by amplification and sequencing of a DNA fragment encoding for a 16S rRNA sequence, according to the manufacturer’s suggestions (Applied Biosystems, USA). The 1500 bp sequence was compared with those present in GenBank using the blastn program, and similarity of 100 % (891 out of the 891 bases recorded in terms of the 16S rRNA) was found with the genome of *Cellulosimicrobium cellulans* strain KH1 (GenBank accession number: AB435177.1). The sequence of the strain was then deposited in the GenBank database with the accession number MN428443.

The MIC values were as follows: erythromycin, 2.0 µg ml^−1^; penicillin, 2.0 µg ml^−1^; vancomycin, 0.5 µg ml^−1^; linezolid, 1 µg ml^−1^; and rifampin, 1.0 µg ml^−1^. Since no Clinical and Laboratory Standards Institute (CLSI) or European Committee on Antimicrobial Susceptibility Testing (EUCAST) breakpoints are provided for this bacterial genus, the Interpretive Standards for the Minimal Inhibitory Concentration (MIC) of *Corynebacterium* provided by EUCAST [1] were used, and the strain was interpreted as being susceptible to vancomycin and linezolid, and resistant to penicillin and rifampin.

**Fig. 1.**
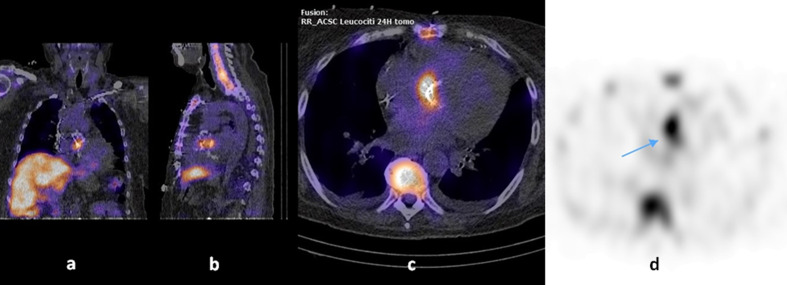
Tc-99m HMPAO WBC SPECT images of a *C. cellulans* aortic prosthetic valve endocarditis. Tc-99m HMPAO WBC SPECT: technetium-99 labelled hexamethylpropylene amine oxime white blood cell single photon emission computed tomography. Fusion coronal (a), sagittal (b) and transaxial (c) scintigraphic tomography/computed tomography images obtained 24 h after administration of the radiolabelled leukocytes. Along with the transaxial (d) scintigraphic tomography image at 24 h, these images show the focal uptake of radiolabelled leukocytes at the right side of the aortic valve prosthesis (arrow).

On day 57 vancomycin and meropenem were shifted to oral linezolid (600 mg q 12 h), but both fever and left leg purpura endured and renal function decreased further (serum creatinine 0.32 mmol l^−1^). The histological examination of a renal biopsy was compatible with an IgA nephropathy with tubulointerstitial nephritis. The patient was diagnosed with an ‘atypical’ presentation of Henoch–Shönlein purpura (i.e. without arthritis and gastrointestinal disease). Prednisone (1 mg/kg/day) was started with improvement of renal function, fever and purpuric lesions. The patient was discharged on day 84 with prednisone 1 mg/kg/day and without antibiotic therapy. The subsequent follow-up ambulatory examinations were unremarkable apart for a progressive decrease of renal function. On day 157 the patient presented again to the emergency room with fever, mental confusion, hypotension and acute left arm ischaemia. A brain CT scan revealed multiple ischaemic lesions and intraparenchymal haemorrhages. A left arm ultrasound evaluation revealed an arterial brachial thrombosis. Blood cultures drawn at admission were negative, and empirical therapy with vancomycin and piperacillin/tazobactam was started. A TEE revealed a possible aortic valve periprosthetic abscess. On day 167 a technetium-99-labelled hexamethylpropylene amine oxime white blood cell single photon emission computed tomography (Tc-99m HMPAO WBC SPECT) was performed (Fig. 1). The scintigraphic findings were interpreted as aortic valve periprosthetic endocarditis with a septic embolism in the left wrist. The patient deceased on day 170. The autoptic examination confirmed the suspected aortic valve periprosthetic abscess and revealed multiple arterial thromboses and septic embolisms in the kidneys, brain, spleen and myocardium. Cultures from the prosthetic valve were not performed.

## Discussion

*C. cellulans* are bacteria belonging to the family *Promicromonosporaceae* (phylum *Actinobacteria *) [2]. They are not acid-fast stained, and despite being Gram-positive, their cells are very readily decolourized. They share some similarities with *Nocardia* spp., as in young cultures a substrate mycelium is produced that later fragments into irregular, curved and club-shaped rods that may be arranged in V forms [2]. Species identification is correctly made by 16S rRNA sequencing. Despite being a widely distributed environmental genus – found in soil, organic matter and aluminum hydroxide anti-acid gel – infections caused by this genus are rarely reported and mainly limited to immunocompromised individuals hosting prosthetic material and other foreign bodies [3, 4]. The taxonomic classification of this genus of coryneform bacteria (former genus: *Oerskovia* and *Cellulomonas *) was recently reassessed based on phylogenetic evidence and chemotaxonomic similarities [5, 6]. This reclassification, along with the technical issues concerning the species identification in clinical specimens and the resulting misinterpretation as skin contaminants – as for other coryneforms – might be partially responsible for the underestimation of the pathogenetic role of these bacteria in human diseases [7]. Endocarditis by *Cellulosimicrobium* spp. is extremely rare [8–11]. There is no standard of care regarding these infections, as the antimicrobial therapy is inferred from the limited clinical experience in *Cellulosimicrobium* spp. bloodstream infections and the *in vitro* antimicrobial sensitivity of the isolate, which is usually sensitive to vancomycin [3, 4]. It is also believed that clinical cure is also dependent on the removal of any infected prosthetic material, as there have been reports of recurrence and persistence of infection even when the target of antimicrobial therapy was presumed to be susceptible *in vitro* [3, 4, 12].

Our patient’s case is similar to the others described in the literature, but shows some peculiarities. Firstly, our patient was not immunocompromised, and her bacteraemia could not be traced to any previous focus of infection. Secondly, despite the clinical suspicion of endocarditis (five minor Duke criteria) [13] and a prolonged and targeted antimicrobial therapy, the negative results of the early echocardiograms led to an underestimation of the clinical significance of her bacteraemia, which was considered to have been cured, and led to the subsequent signs and symptoms of the patient being reclassified as manifestations of an atypical presentation of Henoch–Shönlein purpura. Thirdly, even if cultures from clinical specimens were not performed during the autoptic examination, we speculate that the prosthetic aortic valve endocarditis and its septic embolisms were due to the prolonged *C. cellulans* bacteraemia of our patient, as the continuing presence of the prosthetic valve and the subsequent immunosuppressive therapy were consistent with the chronicization of the endocarditis.

## Conclusion

Species identification in cases of coryneform bacteria isolated from blood cultures is challenging, as they should not be routinely underrated as mere contaminants, but they should be considered to be potential pathogens, even in immunocompetent hosts. *Cellulosimicrobium* spp. endocarditis is extremely rare, and the removal of any prosthetic material along with prolonged *in vitro* active antimicrobial therapy should be pursued in order to reduce persistence or relapses of infection. Tc-99m HMPAO WBC SPECT is a useful tool in the diagnosis of prosthetic valve endocarditis, even when caused by nocardioform bacteria.
